# Lithology and disturbance drive cavefish and cave crayfish occurrence in the Ozark Highlands ecoregion

**DOI:** 10.1038/s41598-022-21791-3

**Published:** 2022-11-15

**Authors:** Joshua B. Mouser, Shannon K. Brewer, Matthew L. Niemiller, Robert Mollenhauer, Ronald A. Van Den Bussche

**Affiliations:** 1 Oklahoma Cooperative Fish and Wildlife Research Unit, Oklahoma State University, Stillwater, OK 74078 USA; 2 U.S. Geological Survey, Oklahoma Cooperative Fish and Wildlife Research Unit, Oklahoma State University, Stillwater, OK 74078 USA; 3 Present Address: U.S. Geological Survey, Alabama Cooperative Fish and Wildlife Research Unit, Auburn University, Auburn, AL 36849 USA; 4 Department of Biological Sciences, The University of Alabama in Huntsville, Huntsville, AL 35899 USA; 5 Present Address: Heart of the Hills Fisheries Science Center, Texas Parks and Wildlife Department, Mountain Home, TX 78058 USA; 6 Department of Integrative Biology, Oklahoma State University, Stillwater, OK 74078 USA

**Keywords:** Ecology, Conservation biology

## Abstract

Diverse communities of groundwater-dwelling organisms (i.e., stygobionts) are important for human wellbeing; however, we lack an understanding of the factors driving their distributions, making it difficult to protect many at-risk species. Therefore, our study objective was to determine the landscape factors related to the occurrence of cavefishes and cave crayfishes in the Ozark Highlands ecoregion, USA. We sampled cavefishes and cave crayfishes at 61 sampling units using both visual and environmental DNA surveys. We then modeled occurrence probability in relation to lithology and human disturbance while accounting for imperfect detection. Our results indicated that occurrence probability of cave crayfishes was negatively associated with human disturbance, whereas there was a weak positive relationship between cavefish occurrence and disturbance. Both cavefishes and cave crayfishes were more likely to occur in limestone rather than dolostone lithology. Our results indicate structuring factors are related to the distribution of these taxa, but with human disturbance as a prevalent modifier of distributions for cave crayfishes. Limiting human alteration near karst features may be warranted to promote the persistence of some stygobionts. Moreover, our results indicate current sampling efforts are inadequate to detect cryptic species; therefore, expanding sampling may be needed to develop effective conservation actions.

## Introduction

Groundwater obligate organisms (hereafter stygobionts^1^) are important to human wellbeing^2^. Diverse stygobiont communities support healthy groundwater ecosystems that humans rely on for drinking water and food production^3–5^. Moreover, some stygobionts are model organisms of evolutionary and human health studies. For example, groundwater amphipods in the genus *Niphargus* have been used to understand evolutionary ecology because of the high variability in their biological and life-history traits and the diversity of habitats in which they are found^6^. Additionally, some cavefishes are model organisms for examining insulin resistance, which has potential implications for diabetes research^7^. Unfortunately, many groundwater species are at risk of extinction.

Stygobiont populations have inherent risks of extinction that are exacerbated by human threats. Many stygobiont species have narrow ranges^8,9^, are long-lived (e.g.^10,11^), reach sexual maturity at a later age (e.g.^12,13^), and lay fewer and larger eggs (e.g.^14–16^)—traits which are often associated with increased extinction risk^17,18^. Additionally, stygobiont persistence is threatened by land-use changes (e.g., agriculture and urbanization), direct human contact (e.g., trampling), habitat loss (e.g., groundwater overexploitation), and climate change, among other threats^2,19,20^. In fact, about 70% of subterranean fauna are listed as threatened, vulnerable, or extinct^21^. Our ability to address these threats and conserve and manage stygobiotic diversity is hindered by limited knowledge of the ecological drivers of stygobiont distributions (i.e., the Wallacean shortfall^2,19,22^).

Occurrence is a fundamental ecological state variable that provides basic information necessary for conservation decisions^23^. Occurrence may be a useful surrogate for abundance when it is difficult or impossible to estimate population sizes (e.g., a high occurrence probability may reflect high abundance^23^), which is common for many subterranean species^2^. In particular, knowing a species’ distribution is useful for directing sampling efforts^24^, predicting how species respond to climate change^25^, calculating invasion or extinction risk^26^, and prioritizing locations for conservation efforts^27^. Ultimately, understanding changes in the abundances and distributions of stygobionts will be critical for mitigating biodiversity loss in groundwater habitats^28^. For example, Domínguez-Domínguez et al*.*^29^ mapped the distribution of Goodeine fishes in Mexico to determine which springs should be protected to promote their persistence.

At least 469 stygobiont species occur in the United States and Canada^21^, but studies examining the factors shaping the distributions of stygobionts have been limited largely to Europe (see^30^ for an overview of these studies). These studies have demonstrated that subterranean species distributions are related to glaciation^31,32^, geology^33^, climate^34^, land use^35,36^, above-ground vegetation^37^, and elevation^38^. These factors may influence stygobiont occurrence specifically or regulate fine-scale features that further define species distributions^39,40^. For example, geology can influence groundwater chemistry, hydrology, and local habitat availability^31,33^.

The Ozark Highlands ecoregion has high stygobiotic diversity^21^, which faces potential threats from human land uses (e.g., the land is 31% and 7% agricultural and urban, respectively^41^). Therefore, our study goal was to identify the relationship between occurrence of cavefishes and cave crayfishes and landscape variables (i.e., land use, elevation, vegetation index, and lithology). We did not include climate and glaciation in our assessment as those factors would be more relevant at coarser spatial scales. Our research can help managers of karst resources prioritize sites for conservation and management efforts and guide efforts to locate new populations in areas with high occurrence probability. Further, our results add to the limited body of knowledge concerning stygobiont distributions in North America.

## Methods

### Study area

**Figure 1:**
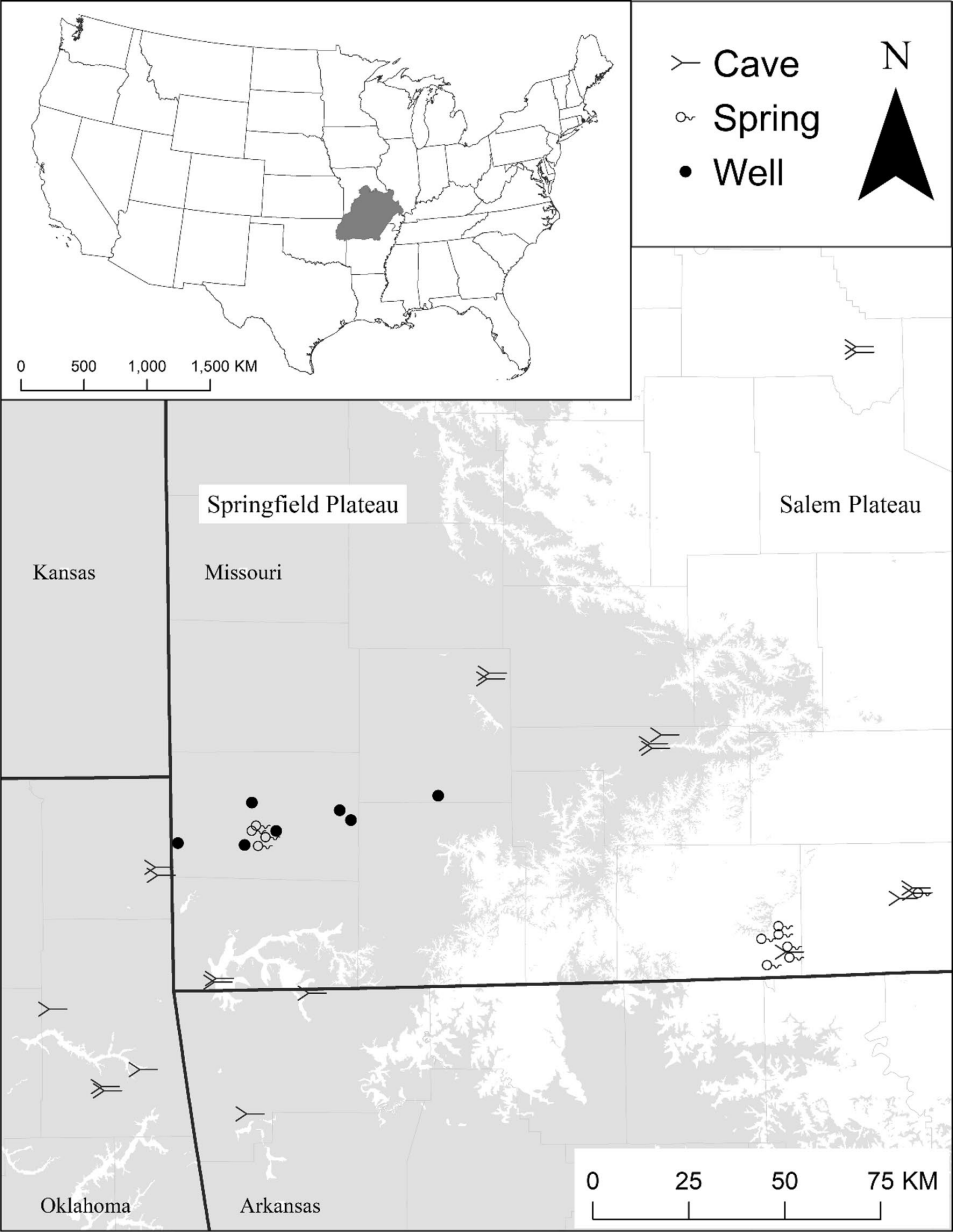
Environmental DNA and visual surveys were conducted for cavefishes and cave crayfishes at 61 sampling units within 21 caves, 12 springs, and seven wells across the Ozarks Highlands ecoregion, USA (dark gray of inset). The lighter gray and white shading on the map represent the Springfield Plateau (i.e., limestone) and Salem Plateau (i.e., dolostone) physiographic regions, respectively. This map was created using ArcGIS software (version 10.4, ESRI, https://www.esri.com/) by Esri. ArcGIS and ArcMap are the intellectual property of Esri and are used herein under license.

We sampled caves, springs, and wells of the Ozark Highlands ecoregion of Missouri, Arkansas, and Oklahoma in the United States (Fig. 1). The Ozark Highlands ecoregion is relatively wet (97–122 cm of precipitation annually) with moderate temperatures (13–16 °C average annual temperature^42^). Many lowland areas have been converted from native, warm-season grasses and oak, hickory, and pine forest to agriculture, whereas many upland areas remain forested^43^. The primary lithologies are limestone and dolomite that through dissolution over time have resulted in cave and spring features emblematic of karst topography^44^.

### Study species

The Ozark Highlands ecoregion is a hotspot of stygobiotic diversity, including snails, copepods, ostracods, amphipods, isopods, amphibians, fishes, and crayfishes^21^. Our study focused on two species of fishes: the Ozark Cavefish *Troglichthys rosae*^45,46^ and Salem Plateau Cavefish *Typhlichthys eigenmanni*^47^ and five species of cave crayfishes: Benton County Cave Crayfish *Cambarus aculabrum*^48,49^, Bristly Cave Crayfish *C. setosus*^50,51^, Delaware County Cave Crayfish *C. subterraneus*^52,53^, Oklahoma Cave Crayfish *C. tartarus*^54,55^, and Caney Mountain Cave Crayfish *Orconectes stygocaneyi*^56^. Although little is known about many of these species, descriptions are quite similar due to convergent evolution (e.g., cryptic behavior, habitat generalists, albinistic, and reduced eyes). Therefore, for our statistical analyses, we treated the species of cavefishes as one taxon, cavefishes, and the species of cave crayfishes as one taxon, cave crayfishes.

### Study design

We conducted both environmental DNA (eDNA) and visual surveys for cavefishes and cave crayfishes at 61 discrete habitat patches (hereafter sampling units) within 21 caves, 12 springs, and seven wells (hereafter sites; Fig. 1). Wells were holes dug at old homesites to access groundwater, caves were underground access to groundwater, and springs were areas where the groundwater met the surface. In most instances, a cave, spring, or well was considered a single site; however, two sites were located in the same cave because they represented two rivers with different hydrologic regimes^57^. We selected one to five sampling units at each site as described by Mouser et al.^58^. Each sampling unit was selected based on hydrologic barriers such as waterfalls or shallow riffles and was separated by at least one habitat patch that was not sampled. For example, wells, springs, and caves with a single pool of water were all considered a single sampling unit, but larger caves with complex habitat had multiple sampling units. Sampling units were surveyed on one to five occasions from February–May 2017 before spring flooding potentially caused changes in species occurrence.

### Species surveys

We collected two water samples (≈ 1-L each) for eDNA analysis following Mouser et al.^58^. Briefly, water was collected from the water column, filtered across 0.45-μm cellulose-nitrate filters, and the filters were stored in Longmire’s buffer^59^. We sterilized sampling equipment by immersion in 50% bleach and rinsing in deionized water. Gear was sterilized between sites and, when possible, between sampling units. We filtered distilled water between sites on four occasions to provide negative field controls. After eDNA collection, two observers walked or crawled the entire sampling unit, while carefully searching the whole wetted area of springs and caves for cave crayfishes or cavefishes following Graening et al.^46,49^. We also visually surveyed hand-dug wells in their entirety for one to six minutes using a spotlight both before and after water samples were collected.

### Detection and occurrence covariates

We selected variables hypothesized to influence stygobiont occurrence and detection probability. We calculated a human disturbance index and recorded dominant lithology associated with each site (i.e., sampling units nested within sites received the same values) to estimate occurrence probability of cavefishes and cave crayfishes. We used land-use data to calculate site-specific human disturbance indices following Mouser et al.^60^. Land-use data were acquired from the 2011 National Land Cover Database^41^. We used ArcMap (version 10.4, ESRI, Redlands, CA) to create 500-m buffers around each site to assess local disturbance. The proportion of each land-use type within the buffers was calculated and multiplied by the following coefficients: open-space development (1.83), low-intensity development (7.31), medium-intensity development (7.31), high-intensity development (8.67), pasture or hay (2.99), cultivated crops (4.54), and undisturbed (1.00, all other categories). The resulting values were summed across all land-use categories to obtain a final disturbance index for each site. We also calculated the difference between the highest and lowest elevation^61^ and the average normalized difference vegetation index (NDVI) for 2016^62^ within the 500-m buffers. Finally, we assigned each site to a lithology category based on the predominant rock type (i.e., limestone or dolostone) within the buffers around each site^63^. To account for variable detection probability, we visually estimated the following covariates for each sampling unit (see^58^ for complete details): water volume (1.0 m^3^), water-column velocity (flowing or not flowing), and substrate (coarse or fine).

### eDNA analysis

We performed eDNA analysis according to Mouser et al.^58^. We designed quantitative Polymerase Chain Reaction (qPCR) Taqman^®^ assays to amplify the DNA of each of the study species. Not all assays developed were species-specific; thus, we sequenced a subset of the positive field samples to confirm species identity. We extracted eDNA from the filters using a Qiagen DNeasy^®^ Blood and Tissue Kit by following the “purification of total DNA from crude lysates” protocol with the modifications found in Mouser et al.^58^. Major modifications included doubling the reagents in steps one to four and decreasing the final elution buffer to 125 μl. We initially extracted a single filter from each sampling unit and our extraction protocol resulted in two subsamples per sampling unit. We amplified eDNA using qPCR. Each subsample was run in triplicate, which resulted in an initial six pseudoreplicates for each sampling unit. We processed the filters until any pseudoreplicates were positive or all were negative for a sampling unit. If any pseudoreplicates were positive, we considered the site positive for the species. We also ran three negative plate controls and a single positive plate control during each qPCR run. The qPCR run was discarded if any of the negative controls amplified.

### Statistical analysis

We used occupancy modeling^64,65^ to estimate occurrence probability of cavefishes and cave crayfishes while accounting for detection probability. Occurrence for taxa *i* at sampling unit *j* was treated as partially observed, with *z*_*ij*_ = 1 if the species was truly present and *z*_*ij*_ = 0 if the species was truly absent. The detection of taxa *i* at sampling unit *j* for survey *k* was conditional on both the true occurrence state and detection probability *p*. Both processes were modeled using a Bernoulli distribution and can be written as:

$$\begin{aligned} z_{ij}&\sim {\text{Bernoulli}}\;(\Psi_{ij} )\\ y_{ijk} &\sim {\text{Bernoulli}}\;(z_{ij} *p_{ijk} ), \end{aligned}$$

where Ψ is occurrence probability.

We modeled variation in Ψ and *p* using linear models^64^. We examined taxa-dependent occurrence probability by allowing each occurrence covariate (i.e., human disturbance and lithology) to vary by taxa. Elevation range and NDVI were not included in the model due to strong correlations with disturbance (Pearson’s pairwise correlation coefficient > 0.65). Lithology and human disturbance were not correlated (point-biserial correlation = − 0.12). Taxa-dependent and sampling-dependent detection probability was modeled by allowing the environmental covariates water volume, water-column velocity, and substrate to vary by taxa and survey method. We also accounted for detection probability by allowing water velocity to vary by survey method. For both models, we used a means parameterization^66,67^ for taxa. This parameterization yields the same model estimates as the dummy variable approach, but provides independent coefficients for each taxon (i.e., the coefficients for the alternate taxa do not represent the difference with reference taxa, but rather the actual estimate). Lithology, survey method, water-column velocity, and substrate were treated as dummy variables with dolostone, eDNA, not flowing, and coarse substrate as reference levels. None of the detection variables were highly correlated^58^. Both water volume and human disturbance were natural-log transformed due to right-skewed distributions. The detection model can be written as:

$$\begin{aligned} {\text{logit}}\;({\text{p}}_{{{\text{ijk}}}} ) &= {{\upalpha }}_{{{\text{1i}}}} + {\upalpha }_{{{\text{2i}}}} X_{{1{\text{jk}}}} + {\upalpha }_{{{\text{3i}}}} X_{{2{\text{jk}}}} + {\upalpha }_{{{\text{4i}}}} X_{{1{\text{jk}}}} X_{2jk} + {\upalpha }_{{{\text{5i}}}} X_{{3{\text{jk}}}} + {\upalpha }_{{{\text{6i}}}} X_{{4{\text{jk}}}} + {\upalpha }_{{{\text{7i}}}} X_{{2{\text{jk}}}} X_{{3{\text{jk}}}}\\ &\quad + {\upalpha }_{{{\text{8i}}}} X_{{2{\text{jk}}}} X_{4jk} ,\quad {\text{for}}\;\; i = {2},\;\; j = {1},{ 2}, \ldots J,\quad {\text{for}}\;\; k = {1},{ 2}, \ldots K, \end{aligned}$$

where *α*_1_ is the taxa intercept, *α*_2_ is the velocity main effect coefficient, *α*_3_ is the survey method coefficient, *α*_4_ is the survey method and velocity interaction coefficient, *α*_5_ is the substrate coefficient, *α*_6_ is the volume coefficient, *α*_7_ is the survey method and substrate interaction coefficient, *α*_8_ is the survey method and volume interaction coefficient, *X*_1_ is velocity, *X*_2_ is method, *X*_3_ is substrate, and *X*_4_ is volume.

Similarly, the occurrence model can be written as:

$${\text{logit}}\;(\Psi_{{{\text{ij}}}} )= {{\upalpha }}_{{{\text{1i}}}} + \beta_{{{\text{1i}}}} X_{{1{\text{j}}}} + {\upbeta }_{{{\text{2i}}}} X_{{2{\text{j}}}} ,\quad {\text{for}}\;\;i = {2},\;\;j = {1},{ 2}, \ldots J,$$

where *β*_1_ is the disturbance index coefficient, *β*_2_ is the lithology coefficient, *X*_*1*_ is the disturbance index, and *X*_*2*_ is lithology*.* All covariates were standardized to a mean of zero and standard deviation of one.

We fit the detection and occurrence models using the program JAGS^68^ called from the statistical software R^69^ using the package jagsUI^70^. We used broad uniform priors for model parameters^67^. Posterior distributions for coefficients were estimated using Markov chain Monte Carlo methods using two chains of 55,000 iterations each after a 5000-iteration burn-in phase and no thinning. We calculated 95% highest density intervals (HDIs; i.e., the probability the true parameter is within the interval) for each coefficient and evaluated plots of the posterior distributions to examine support for relationships with cavefish and cave crayfish occurrence probability. We assessed convergence using the Brooks–Gelman–Rubin statistic ($\hat{R}$^71^). $\hat{R}$ values < 1.1 indicate adequate mixing of chains^70,72^. Model fit was assessed using a Bayesian *p*-value. A Bayesian *p*-value between 0.10–0.90 suggests adequate fit^67,73,74^.

## Results

### Species surveys

Cavefishes were observed in more sampling units than cave crayfishes. We detected Ozark Cavefish (i.e., either positive for the species DNA or a visual confirmation) at 31 of 55 sampling units (24 sites) and Salem Plateau Cavefish at four of six sampling units (two sites) where they are hypothesized or known to occur. The small number of sampling units where Salem Plateau Cavefish was observed was an artifact of only sampling a single cave from its much larger distribution. We detected Ozark Cavefish at 6 sites where they have not been previously detected using eDNA surveys. The Bristly Cave Crayfish had the largest distribution of the cave crayfishes and was observed at 12 sampling units (nine sites) where they are hypothesized or known to occur. We detected Benton County Cave Crayfish at four of six sampling units (two sites), Delaware County Cave Crayfish at one of two sampling units (one site), Oklahoma Cave Crayfish at all 6 sampling units (three sites), and Caney Mountain Cave Crayfish at two of four sampling units (two sites) where they are hypothesized or known to occur. We detected Caney Mountain Cave Crayfish at a site where it had not been previously detected using eDNA surveys. All of the negative controls collected in the field were negative, indicating our decontamination protocol was adequate and the absence of false positives.

### Detection and occurrence covariates

Lithology and human disturbance were included in our analysis as occurrence covariates while using water volume, water-column velocity, and substrate as covariates to account for imperfect sampling detection. Our sampling units were located within limestone (*n* = 43) and dolostone lithologies (*n* = 18). Human disturbance index values ranged from 1.00 to 7.79 (mean ± SD = 2.02 ± 0.99), where 1.00 would represent undisturbed and 8.67 would represent the most highly disturbed via the index. Water volume ranged from 0.6 to 800.0 m^3^ (mean ± SD = 64.0 ± 130.2). One hundred twenty-eight surveys were classified as having flowing water and 105 with water not flowing. Thirty-four sampling units had coarse substrate and 27 sampling units had fine substrate.

### Statistical analysis

**Figure 2:**
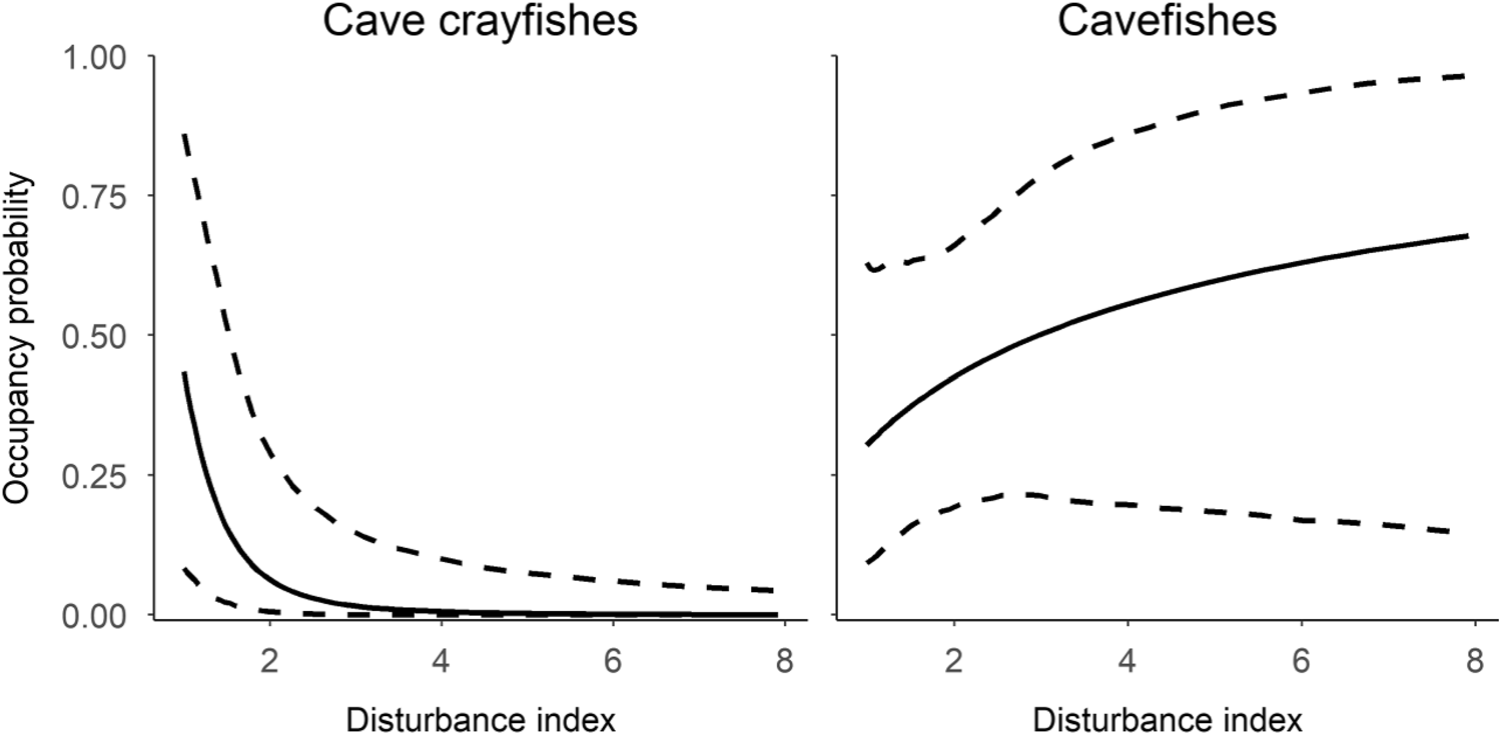
The modeled relationship between human disturbance and occurrence probability of cavefishes and cave crayfishes. Lower numbers along the x-axis indicate less human disturbance, whereas higher numbers indicate higher human disturbance. Solid lines depict the modeled relationship and dotted lines reflect 95% confidence limits (i.e., the uncertainty around the estimated occurrence probability). The categorical variable lithology was set to dolostone.

**Figure 3:**
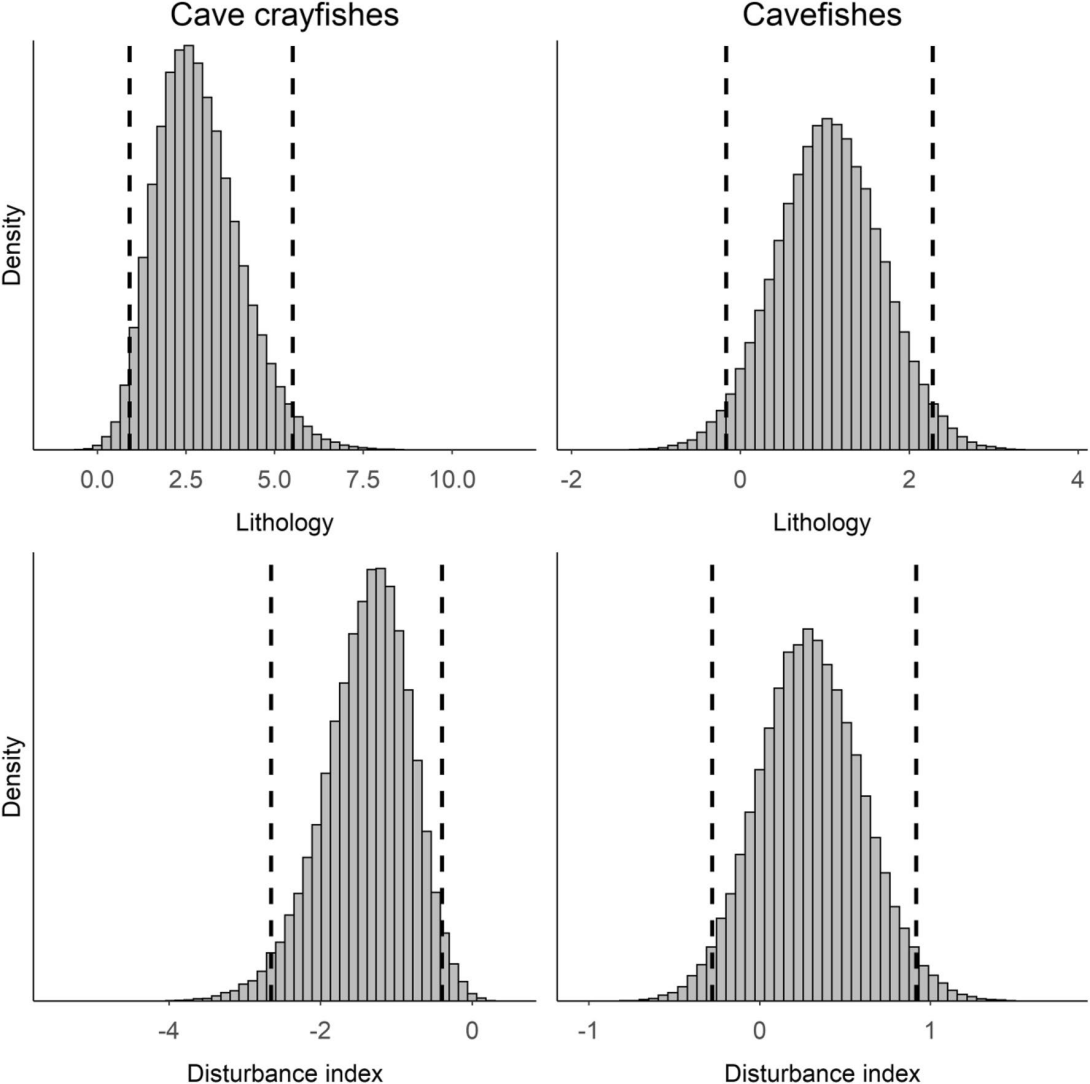
Posterior distributions for the coefficients from our model used to predict occurrence probability of cavefishes and cave crayfishes. The values on the x-axis represent parameter estimates for each covariate. The dotted lines are 95% highest density intervals. The categorical variable lithology was set to dolostone.

Our final model indicated that lithology and human disturbance influenced the occurrence of both cavefishes and cave crayfishes (Table 1, Figs. 2, 3) after accounting for the influence of water volume, substrate, and water velocity on detection probability (Table 2). Both taxa were more likely to occur in limestone compared to dolostone lithology based on the posterior distributions having the greatest density at values than > zero (Fig. 3). Cave crayfish occurrence probability decreased sharply with small increases in human disturbance (Fig. 2). The posterior distribution and 95% HDI for the disturbance slope also supported a strong negative relationship with cave crayfish occurrence (Fig. 3). There was slight evidence of a positive relationship between disturbance and cavefish occurrence based on the posterior distribution (Figs. 2, 3). Detection of cave crayfishes decreased when sampling in locations with higher water volume (Table 2). Cavefish detection decreased when using visual surveys in sampling units classified by fine rather than coarse substrates. Lastly, detection probability of both taxa decreased when using visual surveys in flowing water. Values of $\hat{R}$ indicated adequate mixing of chains. The Bayesian *p*-value was 0.47, which indicated adequate model fit.

**Table 1:** Estimated occurrence probability of cavefishes and cave crayfishes while accounting for imperfect detection derived from an occupancy model.

Parameter	Mean ± SD	95% HDI
Cave crayfish intercept	− 2.45 ± 1.09	− 4.59, − 0.61
Cavefish intercept	− 0.35 ± 0.55	− 1.47, 0.62
Cave crayfish disturbance index	− 1.37 ± 0.57	− 2.59, − 0.36
Cavefish disturbance index	0.30 ± 0.31	− 0.33, 0.87
Cave crayfish lithology	2.83 ± 1.20	0.88, 5.30
Cavefish lithology	1.05 ± 0.64	− 0.25, 2.32

*HDI* highest density interval.

**Table 2:** Estimated detection probability of cavefishes and cave crayfishes in relation to sampling method and environmental covariates derived from an occupancy model.

Parameter	Mean ± SD	95% HDI
Cave crayfish	− 0.17 ± 0.31	− 0.88, 0.42
Cavefish	− 0.08 ± 0.25	− 0.61, 0.47
Velocity	0.74 ± 0.35	0.08, 1.47
Cave crayfish method	0.73 ± 0.57	− 0.32, 1.84
Cavefish method	− 0.67 ± 0.48	− 1.60, 0.28
Method × velocity	− 1.54 ± 0.54	− 2.58, − 0.53
Cave crayfish substrate	− 1.11 ± 0.78	− 2.68, 0.33
Cavefish substrate	0.93 ± 0.41	0.12, 1.71
Cave crayfish volume	− 0.04 ± 0.23	− 0.46, 0.45
Cavefish volume	− 0.11 ± 0.16	− 0.44, 0.17
Cave crayfish substrate × method	0.54 ± 1.04	− 1.40, 2.57
Cavefish substrate × method	− 1.86 ± 0.71	− 3.23, − 0.44
Cave crayfish volume × method	− 1.15 ± 0.39	− 1.85, − 0.36
Cavefish volume × method	− 0.41 ± 0.27	− 0.94, 0.10

*HDI* highest density interval.

## Discussion

We found that occurrence of some Ozark stygobionts had a strong negative relationship with human disturbance, which could be explained by associated physicochemical changes in the groundwater habitat. Higher human disturbance values reflected increased proportions of urban and agricultural land use, which can result in decreased water quality and altered hydrology in surface streams^75,76^. Impaired surface water will likely lead to poor water quality in aquifers because surface streams are the primary recharge sources in the Ozark Highlands^77^. Cavefishes showed a slight positive relationship with disturbance, which is surprising because they are thought to have low tolerance to water quality degradation^46^. In contrast to cavefishes, we found that cave crayfishes had a strong negative relationship with human disturbance. Cave crayfishes may be more influenced than cavefishes by human-induced landscape changes because of pesticide applications in urban and agricultural settings that specifically target arthropods^78,79^. Follow-up testing of the chemical constituents present in runoff may be warranted to better understand mechanisms on species persistence. The relationships observed between stygobiont occurrence and human disturbance, and geology could also be explained by food availability because higher human disturbance would result in less above-ground vegetation and less food availability underground^30^. Other studies have shown that above-ground vegetation (e.g., forest cover) is related to subterranean species occurrence^34,37^.

The lithology associated with our sites also influenced the occurrence of cavefishes and cave crayfishes. In other studies of subterranean species’ distributions, lithology was hypothesized to represent habitat availability, water chemistry, and physical barriers^31,33^. Both lithology types found in our study area represent karst habitat that are chemically and physically capable of supporting stygobionts, so physical barriers between the lithology types could be the actual driver of the relationship. The areas we classified as dolostone lithology correspond roughly with the Salem Plateau, whereas the areas we classified as limestone lithology correspond with the Springfield Plateau (Fig. 1). The hydrogeological differences between the Salem and Springfield plateaus could serve as a physical barrier that limits species to a particular region^80^. However, we detected Ozark Cavefish via eDNA in the Salem Plateau, and they are currently thought to occur only in the Springfield Plateau^46,80^. The cave crayfishes appear to be more isolated by lithology as individual species more closely align with the Salem or Springfield plateaus.

Our results can help guide future conservation efforts for stygobionts. Many populations of stygobionts occur near rapidly expanding cities or near fields used for agriculture, and in some instances, most, if not all, of their known range is threatened by human land use (e.g.^81–83^). We found that cave crayfishes have a strong negative relationship with human disturbance; therefore, limiting agricultural and urban development in karst locations is worth considering if the goal is to protect at-risk populations. Human disturbance can be reduced through the implementation of freshwater protected areas that completely exclude development and are designed specifically to protect key ecosystem processes^84^. When completely excluding development from karst areas is not feasible, agricultural and urban best management practices can be implemented to reduce pollution of groundwater while allowing human activities to continue^85^. We also found that some species might occur outside of their known range. For example, Ozark Cavefish are currently thought to be restricted to the Springfield Plateau^46,80^. However, we detected their DNA in the Salem Plateau. Current sampling efforts are often not adequate to detect cryptic stygobiont species^58^; therefore, expanding sampling efforts might be needed if the goal is to make effective conservation decisions^2^.

We have identified that human changes and lithology play important roles in structuring the distribution of both cavefishes and cave crayfishes. However, cavefishes generally receive more conservation focus than cave crayfishes as indicated by federal listing (i.e., half of cavefishes are federally listed, whereas < 10% of cave crayfishes are listed). Our results indicate that cave crayfishes may be more sensitive to land-use changes than is reflected by their listing status. Cavefishes may be equally sensitive to land-use changes, but our results may have indicated a positive relationship with disturbance because disturbance is positively correlated with other factors that influence cavefish occurrence (e.g., baseflow). Our results indicate that protecting both cavefishes and cave crayfishes begins with improved sampling efforts to understand where these species occur and then protecting populations from human-induced landscape changes.

## Data Availability

The dataset generated and analyzed during the current study is available in the SHAREOK repository [10.22488/okstate.22.000004].
